# Fermentation of Plant-Based Feeds with *Lactobacillus acidophilus* Improves the Survival and Intestinal Health of Juvenile Nile Tilapia (*Oreochromis niloticus*) Reared in a Biofloc System

**DOI:** 10.3390/ani14020332

**Published:** 2024-01-21

**Authors:** Nataly Oliveira Dos Santos Neves, Juliano De Dea Lindner, Larissa Stockhausen, Fernanda Regina Delziovo, Mariana Bender, Letícia Serzedello, Luiz Augusto Cipriani, Natalia Ha, Everton Skoronski, Enric Gisbert, Ignasi Sanahuja, Thiago El Hadi Perez Fabregat

**Affiliations:** 1 Department of Animal Science (Pisciculture), Universidade do Estado de Santa Catarina (UDESC), Av. Luiz de Camões, 2090, Bairro Conta Dinheiro, Lages 88520-000, SC, Brazil; natalyneves1997@gmail.com (N.O.D.S.N.); lari.stock@gmail.com (L.S.); fernandadelziovo@gmail.com (F.R.D.); bendermari0205@gmail.com (M.B.); leserzedello@hotmail.com (L.S.); ciprianiaugustoluiz@gmail.com (L.A.C.); ha.natalia@yahoo.com.br (N.H.); everton.skoronski@udesc.br (E.S.); 2 Department of Food Science and Technology, Universidade Federal de Santa Catarina (UFSC), Rod. Admar Gonzaga, 1346, Bairro Itacorubi, Florianópolis 88034-000, SC, Brazil; juliano.lindner@gmail.com; 3 Aquaculture Program, Institute of Agrifood Research and Technology (IRTA-La Ràpita), Ctra. Poble Nou. Km 5.5, 43540 La Ràpita, Spain; enric.gisbert@irta.cat

**Keywords:** intestinal health, lactic acid bacteria, solid-state fermentation, soybean meal, survival

## Abstract

**Simple Summary:**

This study investigated the effect of *Lactobacillus acidophilus* fermentation on plant-based aquafeed’s biochemical and nutritional profiles, as well as its impact on the productive performance and intestinal health of juvenile Nile tilapia (*Oreochromis niloticus*) reared in a biofloc system. Two fermentation times of 6 h and 18 h were assessed over 60 days and compared with positive and negative control diets containing fishmeal or devoid of animal protein, respectively. *L. acidophilus* fermentation improved the plant-based feed. Fish fed with the diet that was fermented for six hours exhibited improved survival rates. Fermentation worsened feed efficiency and increased feed intake. Fermented feeds positively influenced intestinal health by increasing beneficial bacteria and reducing pathogenic strains in both the rearing water and the fish’s guts. Fermented feeds also enhanced intestinal mucosa development compared to non-fermented diets. These results emphasize the promising impact of aquafeeds fermented with *L. acidophilus* on fish feeds and health and its sustainability by replacing the use of fishmeal with the use of plant protein.

**Abstract:**

This study evaluated the effect of fermentation with *Lactobacillus acidophilus* on the biochemical and nutritional compositions of a plant-based diet and its effects on the productive performance and intestinal health of juvenile Nile tilapia (*Oreochromis niloticus*) reared in a biofloc technology (BFT) system. The in vitro kinetics of feed fermentation were studied to determine the *L. acidophilus* growth and acidification curve through counting the colony-forming units (CFUs) mL^−1^ and measuring the pH. Physicochemical and bromatological analyses of the feed were also performed. Based on the microbial growth kinetics results, vegetable-based Nile tilapia feeds fermented for 6 (FPB6) and 18 (FPB18) h were evaluated for 60 days. Fermented diets were compared with a positive control diet containing fishmeal (CFM) and a negative control diet without animal protein (CPB). Fermentation with *L. acidophilus* increased lactic acid bacteria (LAB) count and the soluble protein concentration of the plant-based feed, as well as decreasing the pH (*p* < 0.05). FPB treatments improved fish survival compared with CPB (*p* < 0.05). Fermentation increased feed intake but worsened feed efficiency (*p* < 0.05). The use of fermented feeds increased the LAB count and reduced pathogenic bacteria both in the BFT system’s water and in the animals’ intestines (*p* < 0.05). Fermented plant-based feeds showed greater villi (FPB6; FPB18) and higher goblet cell (FPB6) counts relative to the non-fermented plant-based feed, which may indicate improved intestinal health. The results obtained in this study are promising and show the sustainable potential of using fermented plant-based feeds in fish feeding rather than animal protein and, in particular, fishmeal.

## 1. Introduction

The production and consumption of fish from aquaculture has been continuously growing since the beginning of the modern aquaculture industry [1]. The intensification of production has led to an increase in the demand for aquafeeds of high nutritional quality. One of the main protein sources of aquafeeds is fishmeal. However, it is an expensive and increasingly scarce product, causing an excessive rise in the prices of aquafeeds [2,3]. The replacement of fishmeal with plant protein sources [4,5,6] has been widely studied in fish diets [2,7,8], but total substitution is not always possible [9,10,11]. In particular, the use of plant-based ingredients in fish diets presents several challenges, such as the presence of antinutritional factors (ANFs), low palatability, and deficiency in the concentration of essential amino acids [12,13,14]. For some species, enteritis and gastric disorders may occur, leading to a reduction in productive parameters [15,16].

Among the different strategies for improving the suitability of plant protein sources for their inclusion at higher levels in aquafeeds, fermentation is one of the most recent ones [6]. During fermentation, microorganisms break down complex substrates, producing metabolites with functional properties [17]. Fermentation is an alternative that can improve palatability, reduce ANFs, and provide functional properties to plant-based ingredients [18]. In particular, several studies have shown that fermentation of plant-based protein sources can improve their nutritional and biochemical quality by reducing ANFs like glycinin and β-conglycinin [12,13,19]. Furthermore, this process may also provide a significant increase in peptides and free amino acid content [18,19,20].

Fermentation can be conducted using different microorganisms, like lactic acid bacteria (LAB), yeasts, and *Bacillus* sp. [21]. The use of LAB enhances the attribution of bioactive properties. Several compounds are produced by LAB during fermentation, such as vitamins, aromatic compounds, antimicrobials like bacteriocins, and antioxidant enzymes [22,23,24]. Microorganisms of the genus *Lactobacillus* are generally recognized as safe for dietary purposes [18,25]. Each strain of LAB can distinctly modify the substrate and the characteristics of the fermented product [26,27]. Within the group of LAB, *Lactobacillus acidophilus* are important microorganisms in the composition of the intestinal microbiota [28]. They are used as a probiotic in fish feed [29], as they exhibit therapeutic activities in intestinal health and produce a variety of antimicrobial compounds that are effective against pathogenic bacteria [30].

Several studies have shown that the use of fermented protein sources with LAB has a positive result on fish growth performance and allows higher inclusions of plant-based protein in the diets [6,13,31,32]. Furthermore, fermented plant-based ingredients have been observed to enhance the antioxidant response [33,34], digestive enzymatic activity, gut condition, and intestinal microbiota [35,36,37,38]. On the other hand, few studies have evaluated the complete substitution of fishmeal with fermented ingredients in plant-based diets [14,39,40]. An approach that has yet to be evaluated is a complete feed fermentation of plant-based diets, which would extend the benefits of fermentation to other ingredients rather than just plant protein sources and optimize feed use. Complete fermentation of plant-based diets may solve issues associated with reduced absorption of micro- and macronutrients in the intestinal tract and improve the nutritional quality and palatability of feeds.

The Nile tilapia (*Oreochromis niloticus*) is one of the most produced and consumed freshwater fish worldwide [25]. It is typically cultured in rustic areas of some countries, is easy to reproduce, and has an omnivorous feeding habit [41]. Recently, the raising of Nile tilapia in biofloc technology (BFT) systems has been developed [42,43]. The BFT system enables increased productivity with low water renewal [42,43,44,45]. It is composed of flocs, which are formed through the conversion of accumulated organic matter, such as feed waste and feces, and the presence of a community of microbial, heterotrophic bacteria, algae, and protozoa [46]. The bioconversion of biofloc flocs is carried out via a system in continuous water aeration, and the addition of a carbon source like molasses to promote bacterial growth [43]. This system enables productivity gains, but microbial growth control can be a problem [47]. The flocs formed are sources of protein, minerals, and vitamins, and serve as a natural food for animals and bacteria [43,44]. Therefore, the use of fermented feeds for tilapia reared in a BFT system could have positive effects on the fish physiology and water microbiota through the interaction between the components of the fermented product and the heterotrophic microorganisms present in the water [47,48,49]. We hypothesize that fermentation of plant-based diets with lactic acid bacteria may improve the productive performance of fish, their intestinal condition, and their intestinal microorganism counts. Thus, this study aimed to evaluate the effect of fermentation with *L. acidophilus* on the biochemical and nutritional composition of plant-based feeds and its effects on water quality, fish performance, and the intestinal health of juvenile Nile tilapia reared in a BFT system.

## 2. Materials and Methods

### 2.1. Experimental Design

This study was divided into two distinct stages. The first stage consisted of several in vitro tests that were carried out at the Laboratories of the Research Group on Food Technology and Bioprocesses at the Federal University of Santa Catarina—Florianópolis, Brazil. The second phase consisted of an in vivo nutritional trial at the Laboratory of Fish Farming of the State University of Santa Catarina at the Center for Agricultural and Veterinary Sciences—Lages, Brazil. The study was approved by the ethics committee for animal use in research (CEUA-UDESC) under protocol No. 8681210822.

### 2.2. Selection of Microorganisms and Their Purity

The strain chosen for fermenting the plant-based feed was *L. acidophilus* (DSM 21717 Coana^®^, Florianópolis, Brazil). This strain was selected based on preliminary pilot tests. Bacteria were reactivated in MRS (Man Rogosa Sharpe, Brand Kasvi, Laboratórios Conda S. A., Madrid, Spain) broth at 36 °C for 48 h before use to verify the purity of the strains. The purity determination was based on morphological evaluation of the strains (*Bacillus*-shaped, green in color, without flagellum) in agar using an optical microscope. After verifying their purity, the strains were stored in 2 mL tubes with a 50% (*v*/*v*) glycerol solution in an ultra-freezer at −80 °C [50,51].

### 2.3. Experimental Feeds

Two isoproteic (33% crude protein) and isoenergetic (4100 kcal kg^−1^) compound feeds with or without fishmeal (FM) were formulated (Table 1) to meet the nutritional requirements of Nile tilapia [52,53], whereas the amino acid composition followed the recommendation given by Santiago and Lovell [54]. The positive control diet (CFM) had 20% FM, whereas the diet containing high levels of plant protein sources (CBP) did not contain FM. Soybean meal was also used as a protein source in both diets, even though its level of inclusion was lower in the CFM (42%) diet when compared to the CBP (65%) diet, while soybean oil, corn, and wheat flour were used as energy sources. All ingredients were ground in an industrial processor, sieved through a 0.71 mm opening mesh, and homogeneously mixed. The mixed ingredients were stored in plastic packages and maintained under refrigeration (4 °C) until pelleting.

The plant-based feed was fermented following a methodology adapted from Azarm and Lee [55]. In brief, autoclaved samples (100 °C for 20 min) of feeds were moistened (30% moisture) with sterile mineral water and inoculated with 2% commercial sucrose and *Saccharomyces cerevisiae* (Fleischmann^®^ dry biological yeast, Heilsbronn, Germany) [40,56]. The *S. cerevisiae* was reactivated in warm water before being inoculated into the feed at a ratio of 60.5 mg for each 2 kg of feed [40]. *S. cerevisiae* was included in a low concentration to serve as a starter for fermentation [56]. Direct inoculum of *L. acidophilus*, previously reactivated, was performed after centrifugation (10 min at 4000 rpm), washing, and resuspension of the pellet at a concentration of Log 8 CFU g^−1^. Samples were mixed and arranged in trays, maintaining a maximum height of two centimeters of feed per tray. The fermentation was carried out in an oven at 36 °C for up to 24 h. At the end of the fermentation process, the fermented feeds were dried in an oven (36 °C) until reaching a constant weight and then kept in a freezer (−20 °C) until their further use.

All feeds were pelleted in a meat grinder with the addition of water (30%) and dried in an oven at 45 °C for 36 h. The drying temperature was low to keep microorganisms active. Previous studies have already shown that LAB in fermented products can remain active after pelleting [39]. The feeds were stored in plastic packages and kept in refrigerators (4 °C) until use.

### 2.4. Characterization of Fermented Feed

Samples of the fermented feed were collected every six hours to count their populations of LAB, measure the pH values, and determine their soluble protein content. A total of 1.6 g of each sample was weighed and diluted in sterile tubes containing sterilized peptone water for the viable cell count (Log CFU g^−1^). Bags were vortexed for ca. 1 min and a serial dilution was performed in the sterilized glass tubes containing 9 mL of peptone water and 1 mL of the sample. Then, 0.1 mL of the selected dilutions was inoculated in triplicate on MRS agar dishes for LAB counting. The dishes were incubated inverted in an oven at 36 °C for 48 h for subsequent CFU mL^−1^ counting [57,58,59]. The pH analyses were performed with 5 g samples, which were weighed and diluted in distilled water. The pH was measured using a benchtop pH meter (Simpla model pH140), with samples being collected every 6 h during the fermentation period [60].

The identification of total soluble proteins was performed using the Bradford method [61]. In brief, 0.002 g of the formulated diets was weighed and added to 2 mL Eppendorf tubes. Samples were diluted in 1 mL PBS solution (1:10, *w*/*v*). The Eppendorf tubes were sonicated (Bandelin Sonopuls HD 2200) for 2.5 min (5 rounds of 30 s with 1 min intervals in an ice bath) for the release of soluble proteins. Then, samples were centrifuged for 10 min at 4000 rpm and their supernatant was used for the Bradford analysis. Subsequently, a 20 mg 10 mL^−1^ solution of BSA (bovine serum albumin) was prepared to generate the standard dilution curve, used for the construction of the calibration graph for the analyses. An aliquot of 10 µL of sample and 1 mL of Bradford’s solution was added to a quartz cuvette. The sample’s optical density was read with a spectrophotometer (Thermo Scientific Genesys 150, Waltham, MA, USA) at λ = 595 nm and the soluble protein (mg g^−1^) levels were determined using the regression equation between the absorbance and protein (BSA) content.

Two fermentation times (6 and 18 h) for the aquafeeds were selected for the test with Nile tilapia to evaluate the effects of total or partial fermentation. In addition to the above-mentioned analyses, bromatological, amino acid composition, leaching rate, and shelf-life analyses were also carried out at the selected times and in the control diets. Proximal composition analyses were performed according to the AOAC [62]. The analysis of the amino acid concentration was performed at the CBO (laboratory analyses CBO, Rio de Janeiro, Brazil) laboratory using the methodology described by White et al. [63] and Lucas et al. [64]. To evaluate the leaching rate, samples (5 g) of feeds were placed in a transparent plastic cup containing 500 mL of water. Two durations of exposure (1 and 3 min) to water were evaluated and after this period, the samples were collected with a sieve, dried in an oven with forced air circulation (55 °C), and weighed. Four replicates were performed for each diet. Based on the difference between the weight of the feeds at the beginning and their weight at the end, the leaching rate was measured.

The shelf life of the diets was determined by evaluating the microbial unfeasibility curve (LAB survival) and mold and yeast counts. For this purpose, the experimental feeds were kept at room temperature and refrigerated. Diets were sampled at 0, 7, 15, 30, and 60 days. A total of 5 g of each sample was weighed on an analytical balance. Three replicates were performed for each diet. After serial dilution, the samples were inoculated in PDA (potato dextrose agar, EP/USP/BAM, Brand Kasvi, Laboratórios Conda S. A., Madrid, Spain) and MRS agar for the quantification of molds and yeasts, as well as LAB, respectively. The PDA dishes were incubated at approximately room temperature for 24 to 48 h. The MRS agar dishes were incubated inverted in an oven at 36 °C for between 24 and 48 h inside anaerobic jars. Counting was performed in triplicates (CFU g^−1^).

### 2.5. Animals and Facilities

A total of 408 masculinized juvenile Nile tilapias with an average initial weight of 8.3 ± 0.2 g (mean ± standard deviation) were provided by a producer located in Pouso Redondo, Santa Catarina (Brazil). After an acclimatization period of 15 days, the fish were distributed in 24 tanks with a useful volume of 70 L at a density of 17 fish per tank. The tanks were equipped with an individual aeration system coupled to a radial compressor. Each experimental unit also had a heater with a thermostat responsible for maintaining a constant water temperature (ca. 26–28 °C) and a temperature-controlled environment at 30 °C. Fish were fed manually twice a day (8 a.m. and 4 p.m.) until apparent satiety. This strategy allowed for the accurate calculation of feed intake for the experimental diets. The tilapias’ avidity to eat allowed for ascertaining their interest in the tested feeds, even in the murky water of the BFT system.

### 2.6. BFT System Maintenance

The rearing tanks were prepared with an inoculation of 20% of water from another mature BFT system (macrocosm). The water in the tanks was slightly salinized (4 g L^−1^) to reduce the fish’s susceptibility to diseases. Salinity was measured using a refractometer (Model RTS-28 Instrutherm). All tanks received uninterrupted oxygenation. The calculation of the amount of organic carbon added to the water was used to maintain the C/N ratio at 15:1, aiming to maintain the heterotrophic system of the biofloc [65,66]. This methodology assumes that fish assimilate approximately 25% of the nitrogen in their food and that the remaining 75% is converted into total ammonia nitrogen (TAN) in the water [67]. The amount of nitrogen was monitored based on an analysis of ammonia in the water using a rapid analysis kit (Labcon Teste^®^, Dois Vizinhos, Brazil), and molasses was added whenever the values exceeded 0.5 mg L^−1^. Sedimentable solids were determined using an aliquot of water collected from each tank and transferred to an Imhoff cone, then evaluated after 20 min. Partial water changes were performed whenever the sedimentable solids exceeded 25 mg L^−1^.

### 2.7. Water Quality

Water quality parameters such as the temperature, pH, dissolved oxygen, ammonia, nitrate and nitrite levels, and sedimentable solids were measured every two days. An Alfakit™ AT170 oximeter (Florianópolis, Brazil) was used for the measuring of the dissolved oxygen and temperature. An Alfakit™ AT100P photocolorimeter (Florianópolis, Brazil) was used to determine the ammonia, nitrate, and nitrite concentrations. Sedimentable solids were determined using an aliquot of water collected from each tank and transferred to an Imhoff cone. The amount of sedimented solids in the cones was evaluated after 20 min of collection. Water samples were collected weekly to perform total suspended solids (TSSs) analyses [68].

The water quality parameters remained within the recommended limits for tilapia rearing in BFT systems [69], as described in Section 3.3. Furthermore, water samples were collected to count the heterotrophic microorganisms, LAB, and *Vibrio* sp. on days 0, 7, 15, 30, and 60. Petri dishes were incubated in an oven at 36 °C. Colony-forming units (CFUs) were counted after 24 h of incubation in TSA (tryptone soy agar, Brand Kasvi, Laboratórios Conda S. A., Madrid, Spain) and TCBS (thiosulfate, citrate, bile, and sucrose agar, Brand Neogen Corporation, Lansing, MI, USA) media and after 48 h in MRS medium.

### 2.8. Fish Performance

At the beginning of the experiments and at 30 and 60 days, all fish were fasted for 24 h, anesthetized with eugenol (50 mg L^−1^), and individually weighed. Productive performance was analyzed based on the following key performance indicators: body weight gain (WG) and apparent feed conversion (FC). Mortality was also recorded to assess the survival rate (SR).

(1) $$Weight\;gain\left(WG\right)=final\;mean\;weight-initial\;mean\;weight$$

(2) $$Apparent\;feed\;conversion\left(FC\right)=\frac{feed\;intake}{total\;weight\;gain}$$

(3) $$Survival\;rate\;(SR)=\frac{total\;animals\;at\;the\;end}{total\;animals\;at\;the\;beginning}\times 100$$

Four fish from each replicate tank were anesthetized and then euthanized via medullary injection at 30 days to count their intestinal microorganisms. Before collection, the fish were fasted for 24 h. On the 60th day of the experiment, biological materials were collected from eight more fish from each experimental unit following the protocol described above: namely, two fish for the intestinal histomorphometry analyses (n = 48), three fish for the intestinal microorganism count analyses (n = 72), and three fish for the analyses of intestinal enzyme activity (n = 72). A pool was created with three fish from each tank for the microorganism count and enzymatic activity analyses, resulting in one sample per experimental unit (n = 24). This strategy was adopted to reduce variability, considering the tank as an experimental unit.

### 2.9. Intestinal Microorganism Count

The intestines of Nile tilapia were aseptically removed, weighed to the nearest 0.1 g, ground, homogenized, and serially diluted (1:10) in test tubes containing sterile saline solution (0.65%). Then, the intestinal homogenates were seeded in Petri dishes with MRS agar, TSA, and TCBS agar to quantify the LAB, total heterotrophic bacteria, and *Vibrio* sp., respectively [57,59,70]. Intestinal homogenates seeded in Petri dishes were incubated in an oven at 35 °C. Colony-forming units (CFUs) were counted after 24 h of incubation in the TSA and TCBS media and after 48 h in the MRS medium.

### 2.10. Intestinal Histomorphometry

Two fish from each replicate per treatment (n = 48) were selected for intestinal histomorphometric analysis after euthanasia. Their intestines were removed and weighed after sectioning the abdominal wall. A portion of the anterior intestine (ca. 4 cm) of each animal was collected and fixed in 15% neutral buffered formaldehyde. Samples were dehydrated in ethanol solutions of increasing concentrations, cleared with xylene, and embedded in paraffin. Serial sections (4 μm thickness) were obtained with a microtome and stained with hematoxylin and eosin for general description, and the periodic acid–Schiff (PAS) method was used for goblet cell identification and counting. Slides were analyzed using an optical microscope, the software ToupTek ToupView ×64 image analyzer version 2270/07/03, and a digital camera (Moticam 2300, 3 MP, resolution of 3264 × 2448, Germany, RML 5). The intestinal villi were evaluated and measured in terms of height, width, thickness, and the total number of goblet cells counted, as described in De Mello et al. [71].

### 2.11. Biochemical Analyses

A pool of three fish from each replicate per treatment were selected for biochemical analysis after euthanasia (total of n = 24 samples; n = 6 samples per treatment). Alpha-amylase, total alkaline protease, and lipase activities were determined according to the methodology described by García-Carreño [72]. The intestines of three fish were collected per tank replicate. During this collection, the samples were immediately frozen in liquid nitrogen (−196 °C) and stored in an ultra-freezer at −80 °C until analysis. To perform the analyses, the samples were then thawed, weighed, and fractionated into smaller sizes to facilitate their homogenization in order to carry out the analysis. They were homogenized using ice-cold sterilized distilled water (1:6 *w*/*v*) and sonicated under refrigeration (0–4 °C) for 5 min. Then, the homogenate was centrifuged at 11,000× *g* for 10 min at 4 °C, and the pellet containing cell debris was discarded. The recovered supernatant was used to define the enzymatic activity using standard protocols. 

Amylase activity was determined through a starch hydrolysis test after interrupting the reaction by using dinitrosalicylic acid with an absorbance at λ = 540 nm, being expressed in moles of reducing sugars [73]. Total alkaline protease activity (U) was evaluated through the quantification of azocasein hydrolysis [72,74]. The lipase concentration was determined using p-nitrophenyl laurate and propanol, and the resulting reaction with the supernatant was stopped using ketone [75]. Lipase activity was measured at a wavelength of λ = 410 nm. The concentration of enzyme required to hydrolyze the lipid components was expressed as lipase activity (U). Samples were kept refrigerated during these analyses and all digestive enzyme determinations were performed in triplicate per sample (methodological replicate). The activity of the digestive enzymes was expressed as specific activity (U mg^−1^ protein).

Regarding oxidative stress enzymes, catalase (CAT) activity was measured by decreasing the absorbance at λ = 240 nm (Ɛ = 40 mM^−1^ cm^−1^) using 50 mM H_2_O_2_ as substrate [76]. Glutathione S-transferase (GST) activity was tested following the formation of the glutathione chlorodinitrobenzene adduct at λ = 340 nm (Ɛ = 9.6 mM^−1^ 1 cm^−1^), using 1 mM 1-chloro-2,4-dinitrobenzene and 1 mM glutathione (GSH) as substrates [77]. Glutathione reductase (GR) activity was determined by measuring NADPH oxidation at λ = 340 nm (Ɛ = 6.22 mM^−1^ cm^−1^), using 20 mM glutathione disulfide and 2 mM NADPH as substrates [78]. The superoxide dismutase (SOD) activity was measured at λ = 550 nm as the degree of inhibition of cytochrome C reduction by O_2_ generated by the xanthine oxidase/hypoxanthine system [79]. The reaction mixture consisted of 50 mM sodium phosphate buffer pH 7.8, 0.1 mM Na 2 EDTA, 50 µM hypoxanthine, 10 µM cytochrome C, and 0.6 U mL^−1^ xanthine oxidase. A unit of SOD activity was defined as the amount of sample causing a 50% inhibition of cytochrome C reduction compared to the baseline record obtained with buffer instead of sample.

The soluble proteins from crude enzyme extracts used for digestive enzyme and antioxidative stress enzyme determination were quantified through the Bradford method [61] using bovine serum albumin, as is standard. Enzymatic activities were expressed as specific enzymatic activity in nmol of catalyzed substrate per milligram of protein (nmol mg^−1^ protein) for the CAT, GST, and GR activities, and in U mg^−1^ protein for the SOD activity. All assays were performed in triplicate at 25 °C, and the absorbance was read using a spectrophotometer (Tecan TM infinite M200, Tecan Trading AG, Männedorf, Switzerland).

### 2.12. Statistical Analyses

Our data are expressed as the mean ± SD. The data were subjected to tests to verify the normality of errors (Shapiro–Wilk) and the homoscedasticity of variances (Levene). Data expressed as percentage values were subjected to arcsine transformation. Differences between the results were determined via analysis of variance (ANOVA), using Tukey’s test at a 5% probability, and linear regressions were carried out with the software Statistic version 10.0.

## 3. Results

### 3.1. Characterization of the Fermented Feed

**Figure 1:**
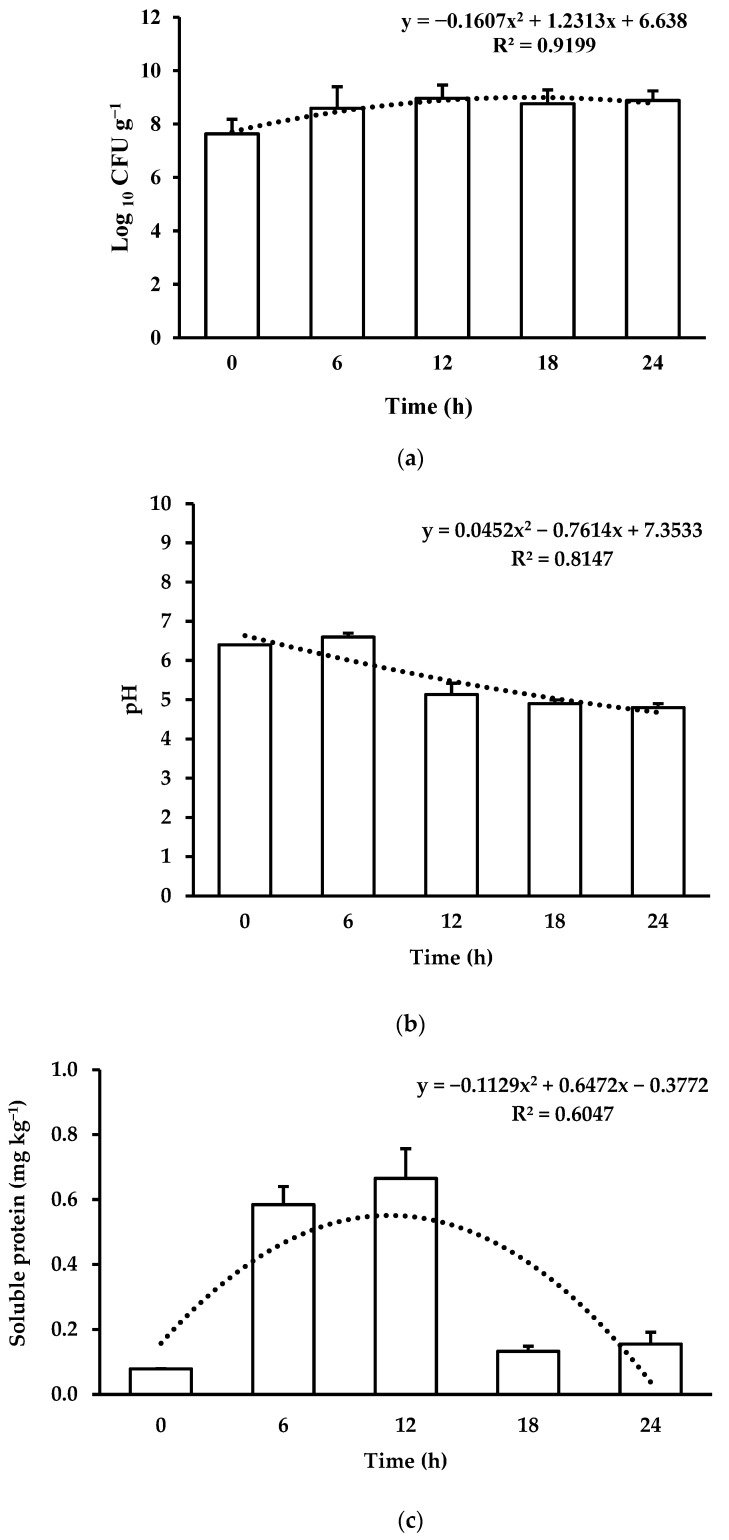
Characterization of plant-based feed fermented with *Lactobacillus acidophilus* at different fermentation times: (**a**) lactic acid bacteria count; (**b**) pH; and (**c**) soluble protein concentration.

The growth kinetics of *L. acidophilus* during the feed fermentation showed a quadratic effect (Figure 1a; *p* < 0.05). The exponential phase of fermentation (Log phase) was reached during the first 6 h of fermentation. LAB CFUs reached their maximum levels after 12 h (stationary phase) and bacterial growth stabilized until the end of the evaluated period (24 h). LAB multiplication reduced the pH in a quadratic way (Figure 1b; *p* < 0.05). There is also a quadratic effect (*p* < 0.05) for the concentration of soluble proteins, showing an increase during the fermentation time (Figure 1c). However, a reduction in the soluble protein concentration was observed after 12 h of fermentation. Fermentation of the feed did not affect its proximate composition (Table 2) nor its amino acid profile (Table 3) (*p* > 0.05).

### 3.2. Leaching Rate and Shelf Life

The leaching rate was higher (*p* < 0.05) for the FPB6 feed after the two evaluated times when compared to the rest of the tested feeds (Table 4). Fermentation increased the shelf life of the fermented plant-based feeds (Table 5). In particular, a significant reduction in the count of molds and yeasts was observed in the FPB6 and FPB18 feeds compared to the two controls at both evaluated times (*p* < 0.05). The lactic acid bacteria also remained viable in the fermented feeds.

### 3.3. Microorganism Count in the Water

Throughout the whole experiment, the water parameters were maintained stable: temperature, 27.09 ± 0.36 °C; pH, 8.40 ± 0.28; dissolved oxygen, 8.08 ± 0.23 mg L^−1^; ammonia, 0.47 ± 0.27 mg NH_3_ L^−1^; nitrite, 0.35 ± 0.09 mg NH_3_ L^−1^; nitrate, 1.43 ± 1.14 mg NH_3_ L^−1^; sedimentable solids, 7.96 ± 3.16 mg L^−1^; and TSS, 225.22 ± 82.08 mg L^−1^. After 60 days of the experiment, the use of FPB18 increased (*p* < 0.05) the LAB count in the BFT tanks compared to the CFM and CPB diets (Table 6). Moreover, a reduction (*p* < 0.05) in *Vibrio* sp. was observed in the water of the tanks that received FPB6 relative to the CFM diet.

### 3.4. Fish Performance

**Figure 2:**
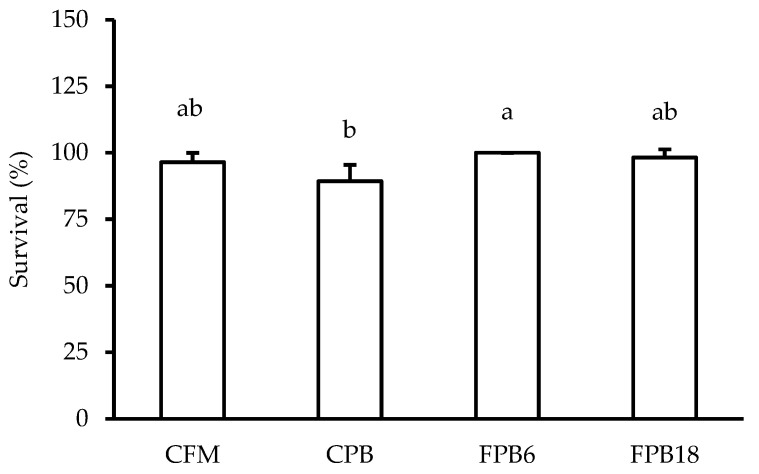
Survival rates of juvenile Nile tilapia (*O. niloticus*) fed experimental diets that differed in their level of fishmeal inclusion (CFM and CPB) and the fermentation time of plant protein feeds (FPB6 and FPB18) after 60 days of the experiment. Percentages followed by different letters differ (ANOVA, *p* < 0.05) from each other according to Tukey’s test. CFM—animal protein feed; CPB—plant-based protein feed; FPB6—6 h fermented plant-based protein feed; FPB18—18 h fermented plant-based protein feed.

The FPB6 feed improved (*p* < 0.05) fish survival compared to the CPB diet (Figure 2). Fermentation of the feed increased (*p* < 0.05) the plant-based feed intake in comparison to the CPB diet (Table 7). The amount of feed ingested by fish from the FPB6 and FPB18 treatments did not differ from the positive control CFM (*p* > 0.05). Regardless of fermentation time, the fermented feeds resulted in worse feed conversion ratios when compared to the non-fermented feeds (*p* < 0.05).

### 3.5. Intestinal Microorganism Count

At the end of the trial, the concentration of heterotrophic bacteria in the intestines of Nile tilapia fed the FPB6 diet was higher than in the rest of the dietary groups (*p* < 0.05). The levels of intestinal LAB at 30 and 60 days were lower (*p* < 0.05) in the fish that received plant-based feeds (Table 8). The count of potential pathogenic microorganisms of the Vibrionaceae family was lower (*p* < 0.05) in the fish treated with fermented plant-based feeds.

### 3.6. Intestinal Histomorphometry

Small intestine epithelial organization was normal and did not show any sign of inflammation in fish from the CBP, FPB6, and FPB18 groups, showing that these treatments were innocuous to epithelium integrity and organization. The villus height of fish that were fed the fermented diets did not differ from their congeners fed the CFM diet (Table 9; *p* > 0.05). The smallest villus size was observed in the fish fed the CPB diet. The number of goblet cells was higher in fish fed the FPB6 diet compared to those given non-fermented feeds. The lowest goblet cell densities were observed in the CPB and CFM groups (*p* < 0.05).

### 3.7. Intestinal Enzymatic Activity

The activities of the α-amylase, lipase, and total alkaline proteases, and the activity of the oxidative stress enzymes, were not affected by the experimental diets (Table 10 and Table 11, respectively; *p* > 0.05).

## 4. Discussion

By means of fermentation, LAB multiply and produce different types of metabolites [80]. Under the current experimental conditions, LAB reached their maximum concentration after 12 h in the fermented feeds. There are no reference values for the growth of LAB during the fermentation of vegetable feeds for Nile tilapia, but the values obtained here are similar [81] or higher [82] than those obtained in the fermentation of soybean substrates for *L. acidophilus.* A reduction in pH and an increase in soluble proteins and cell growth were also observed in the first 12 h of fermentation, which may be a consequence of organic acid production by LAB [83], and because of an accumulation of peptides and low-molecular weight proteins resulting from the fermentation process [18], since LAB break down proteins, releasing peptides [80]. Another hypothesis for the variation in the concentration of soluble proteins could be the reduction in pH during fermentation, which can alter the solubility of the proteins present in the feed [84]. Similar results were also observed during the fermentation of soybean meal with LAB and in combination with other microorganisms [39,82]. In addition, as peptides are an available substrate for bacterial growth [18], this may explain the faint growth of LAB under the current fermentation conditions. 

Fermentation did not affect the bromatological composition and amino acid profile of the feeds. Crude protein values remained constant in all treatments, which fits the nutritional requirements of juvenile Nile tilapia [53]. Studies with soybean meal fermentation have shown that there may be an increase in proteins and amino acids, but that these levels are maintained in most cases [39,85]. Protein synthesis during fermentation may not occur, wherein only the hydrolysis of larger proteins, releasing peptides and free amino acids, takes places [86,87,88]. The increase in soluble proteins confirms the higher availability of low-molecular weight peptides, which may be easily absorbed and used by the host [31,39,89]. However, more studies are still needed to characterize the peptide profile in fermented feeds and their functionality, as well as their impact on fish condition, health, and growth. Under our experimental conditions, the leaching rate increased in feeds fermented for six hours. This result may also be related to a higher concentration of soluble proteins, which may have reduced stability and led to nutrient losses. Factors such as the choice of fermenting microorganism, substrate, time, and temperature can influence the levels of amino acids and the nutritional quality of the feed during fermentation and can be used to adjust the nutritional quality of fermented feeds according to the stage of development and nutritional requirements of farmed fish [85,90,91].

The shelf-life analyses of our fermented feeds showed that the LAB remained viable even after pelleting the feed. The fermented plant-based feeds had the lowest count of molds and yeasts compared to the two controls over the 60 days of evaluation. The increased shelf life of these fermented products can be associated with the production of antimicrobial substances and the pH reduction, having probiotic effects on the host and protecting feed spoilage [92]. Bacteria of the genus *Lactobacillus* may release antimicrobial peptides such as exogenous enzymes (lysozyme) and bacteriocins under situations of competition or stress [93,94]; these substances inhibit the spoilage microflora, such as molds and fungi, present in food [94]. The release of organic acids, such as lactic and acetic, during fermentation by LAB can also be an inhibitory factor for the growth of spoilage bacteria [95]. Organic acids exhibit antifungal activity by interacting with the cell wall of the microorganism [95]. Potential future studies characterizing the bacteriocins and organic acids present in fermented feeds are important to better understand these results and the further application of fermented feeds.

The water quality dynamics of the BFT system were not affected by the administration of fermented feeds. According to Mohammadi et al. [96], the recommended values of TSS for Nile tilapia cultivation in BFT systems should remain between 300 and 690 mg L^−1^, which are considerably higher than the values found in the present study for this species. Furthermore, heterotrophic bacteria growth was effective in controlling ammonia levels and the cycling of nitrogenous compounds, as demonstrated by the low levels of nitrite and increased nitrate. Additionally, the use of fermented feeds positively affected the count of microorganisms in the water in the BFT tanks, with an increase in LAB and a reduction in *Vibrio* sp. The increase in LAB during fermentation may have contributed to the reduction in *Vibrio* sp., through the competitive exclusion process [97]. The use of probiotics has already been demonstrated to be effective in BFT systems to control pathogenic bacteria in water [98]. The presented results on water microbiota modulation with the use of fermented feeds are unprecedented and demonstrate the double benefit of implementing this strategy in BFT systems, since not only are the shelf lives of the feeds extended, but also the water quality is improved.

Survival of these Nile tilapia juveniles was higher in the fish fed with the FPB6 diet compared to the plant-based diets. This result is unprecedented with fermented feeds and ingredients. The use of fermented soybean meal in fish diets has shown evidence of improvement in intestinal health, but survival is generally not affected [99,100,101]. In a recent study, the use of a fermented feed with a co-culture of LAB did not affect the survival of largemouth bass *Micropterus salmoides* [102]. By expanding the benefits of fermenting the complete feed, the effects of fermenting were able to be maximized. The improved survival may be linked to an increase in both the LAB concentration and the metabolites produced in fermentation and the probiotic effect of LAB on the host. In this sense, fermentation led to an exponential growth of *L. acidophilus*, which has probiotic properties [103] and produces substances with nutraceutical properties, improving the intestinal health of the fish and its overall condition [104]. Several studies have evidenced that the direct use of LAB in tilapia feeds can increase their survival [105,106,107]. The decrease observed in the *Vibrio* sp. in the water of the treatments with fermented feeds may also explain their better survival. More evaluations under challenge conditions are required to evaluate the nutraceutical potential of fermented feeds and test their use as functional diets.

In general, feeds containing animal protein (CFM) are more ingested by Nile tilapia [108]. In the present study, the ingestion of fermented plant-based feeds did not differ from the control diet containing fishmeal. This result confirms the potential of feed fermentation as a strategy to improve the palatability of plant-based diets. Aromatic organic compounds are released during the fermentation process, improving the palatability of feeds, and promoting fish ingesta [109]. This is the first study conducted with fermented plant-based feeds, but it has already been demonstrated that soybean meal fermented with LAB can partially replace fishmeal without impairing feed intake and fish performance [100,101,102,103,104,105,106,107,108,109,110]. This result, however, may vary for different species. For example, fermentation of conventional diets for largemouth bass reduced their ingestion rate [102]. Regarding the current study’s promising results, more studies are still needed to identify the palatability enhancers present in fermented feeds and evaluate their effect on fish feeding behavior.

Fermentation of the vegetable feeds had no positive effect on fish weight gain, and the best growth was obtained with the CFM diet. Fermented feeds showed a worsening of feed conversion, which may have harmed the results in terms of production performance. However, no leftover feed was found in any treatment and there were no changes in the suspended solids and water quality that could indicate an increase in bioflocs due to the decomposition of unconsumed feed by heterotrophic bacteria in the tanks. Thus, the worsening in the food conversion values can be explained by the presence of antinutritional factors from vegetable protein sources in the fermented feeds. Soybean meal contains allergenic proteins (β-conglycinin and glycine), insoluble fiber, phytates, and tannins, which may not have been completely eliminated during fermentation [111,112,113,114]. Furthermore, the feed fermented for 6 h had a higher concentration of soluble proteins, which may have led to an excess of low-molecular weight peptides and free amino acids that could compromise their absorption and subsequent use [115,116]. Similar results have been obtained with the inclusion of high levels of fermented soybean meal in diets of catfish (Rhamdia quelen) [82]. Furthermore, changes in the leaching rate that may affect dietary protein levels may also explain the poorer conversion of this diet. Thus, the effects on feed conversion need to be better understood to make fermentation viable as a sustainable practice.

Regarding the bacteria counts in the guts of these Nile tilapia fed experimental diets, an increase in the count of heterotrophic bacteria and LAB was observed in the intestines of the fish with the use of fermented feeds. The intestinal microbial count parameter is an indication of fish health [117]; thus, this increase in intestinal LAB agrees with the increase in LAB count in the feeds and water samples, since there is generally a sound correlation between the intestinal microbiota in fish and those of the environment [118]. A similar modulation effect could have been observed in the reduction in *Vibrio* sp. through the feed and biofloc particles. In addition, LAB have been described to phagocyte pathogenic bacteria and produce immunostimulant and antimicrobial substances [119,120], which may explain the above-mentioned reduction in *Vibrio* sp. abundance. Similarly, the ingestion of soybean meal fermented with *L. acidophilus* has been reported to reduce the count of *Vibrio* sp. in the intestines of catfish [84].

The morphometric characteristics of the tilapia epithelium fed the FPB6 and FBB18 diets were similar to those fed the CFM diet. In relation to the plant-based diet (CPB), there was a reduction in villus height, which can be attributed to the presence of antinutritional factors in soybean meal, such as insoluble fibers and antigenic proteins [121,122,123,124,125]. However, dietary fermentation could have somewhat offset the aforementioned effects of the plant-based diet. These results can be attributed to an improvement in intestinal condition due to the probiotic effect of fermented aquatic feeds and their greater abundance of LAB. Furthermore, dietary LAB has been described to positively modulate intestinal mucosa and villous condition [123]. For example, the use of plant-based diets with fermented soy for tilapia showed an increase in this parameter compared to a diet containing fishmeal [39]. Regarding the intestinal goblet cells, their number was greater in the fish fed the FPB6 diet. These secretory cells lining the intestinal epithelium are responsible for the production of mucins [124] associated with intestinal health and protection [71], as well as non-specific intestinal immunity [125]. Increased goblet cell concentration may be associated with improved gut health, supported by increased beneficial bacteria counts and increased villus size, results that may be linked to the potential probiotic effect of fermented feeds. In this regard, several studies have revealed that consumption of foods containing LAB can increase the number of goblet cells in the intestines of Nile tilapia due to the probiotic effect of LAB [126]. Similarly, different levels of fermented soy in aquafeeds for Nile tilapia resulted in an increase in goblet cell density [39]. 

In this study, fermentation of plant-based feeds did not modulate the activity of digestive and antioxidant enzymes in the gut of Nile tilapia. During fermentation, digestive and antioxidant enzymes are produced by *L. acidophilus* [22,82,127], although their contribution to the host’s condition is doubtful. Dietary inclusion of fermented ingredients may improve the activity of digestive [35] and antioxidant enzymes in fish [128], but changes in these activities are not always observed [82,102,129]. In this study, it is worth highlighting that enzymatic activity was not negatively affected by any treatment, even by the CPB diet, especially when considering that soybean meal may contain enzyme inhibitors that could have negatively affected the digestive process [130] and antioxidant response [131].

## 5. Conclusions

Fermentation with *L. acidophilus* increased the LAB count and soluble protein concentration of the plant-based feed, as well as causing a decrease in pH. The FPB6 diet increased fish survival. Fermentation also increased feed intake but reduced feed efficiency. In general terms, the use of fermented feeds like the FPB6 and FPB18 diets increased the abundance of beneficial bacteria (LAB) counts and reduced the pathogenic bacteria in the water of the BFT system and in the fish’s guts. Fermented diets could promote intestinal conditions by inducing an increase in villus size (FPB6 and FPB18) and goblet cell number (FPB6). The results of this study are promising and show the potential of using plant-based fermented feeds in fish feeding. Fermentation was successfully conducted, and promising results were obtained for the intestinal microorganism count and histomorphometry. This is a promising technology that can be applied both before and after processing and could potentially be used in the production of sustainable fish feed without components of animal origin. Fermentation is a process that can be carried out easily, enabling the adoption of this technology by fish producers, who could apply it to already-processed feeds, reducing production costs. There is a demand for research and development in this field, as it is a new topic with potential impact in fish aquaculture. The prospect of different microorganisms and the effects of fermented feeds on different types of pathogens still need to be evaluated.

## Figures and Tables

**Table 1:** Composition of experimental diets containing different levels of fishmeal and soybean meal for Nile tilapia (*Oreochromis niloticus*).

(%) Ingredient	CFM	CPB
Soybean meal	42.0	65.0
Fishmeal	21.7	-
Corn	19.5	20.0
Wheat bran	13.0	13.0
Soybean oil	3.0	1.0
Premix *	0.5	0.5
DL-methionine	0.30	0.5
Total (%)	100	100
Calculated composition		
DM (%)	89.21	88.15
CP (%)	33.64	33.83
GE (kcal kg^−1^)	4116.07	4131.11
EE (%)	7.34	5.03
CF (%)	4.87	6.41
MM (%)	11.16	4.90

CFM—animal protein feed; CPB—plant-based protein feed; DM—dry matter; CP—crude protein; GE—gross energy; EE—ether extract; CF—crude fiber; MM –mineral matter. * Premix—folic acid: 1000 mg kg^−1^, calcium pantothenate: 9000 mg kg^−1^, biotin: 100 mg kg^−1^, vit. A: 2,400,000 IU, vit. D3: 48,000 IU, vit. E: 24,000 IU, vit. B1: 1600 mg, vit. B2: 9600 mg, vit. B6: 2,600,000 IU kg^−1^, vit. B12: 4000 IU kg^−1^, vit. K3: 609 mg kg^−1^, vit. C: 49 g kg^−1^, iron: 20 g kg^−1^, manganese: 5980 mg kg^−1^, zinc: 28 g kg^−1^, BHA: 196 mg kg^−1^, BHT: 3040 mg kg^−1^, iodine: 200 mg kg^−1^, selenium: 60 mg kg^−1^, niacin: 36 mg kg^−1^, choline: 60 g kg^−1^, inositol: 2000 mg kg^−1^ (minimum guaranteed levels per kg of product). The FM was purchased at Agroforte^®^ (Laguna, Santa Catarina, Brazil; crude protein = 58.8%), the multivitamin premix was provided by Quimtia^®^ (Lima, Perú) and the other ingredients were purchased from local suppliers.

**Table 2:** Proximate composition analyses (% in dry weight) of the experimental diets tested in Nile tilapia (*O. niloticus)* reared under biofloc technology (BFT).

	CFM	CPB	FPB6	FPB18
Dry matter (%)	95.39 ± 0.08	94.78 ± 0.02	94.78 ± 0.07	93.84 ± 0.19
Mineral material (%)	12.8 ± 0.06	5.48 ± 0.04	5.42 ± 0.01	5.47 ± 0.11
Crude protein (%)	34.14 ± 1.90	36.89 ± 1.94	35.88 ± 1.92	36.57 ± 1.93
Ether extract (%)	3.7 ± 3.42	5.77 ± 0.09	3.64 ± 2.92	4.07 ± 1.71

CFM—animal protein feed; CPB—plant-based protein feed; FPB6—6 h fermented plant-based protein feed; FPB18—18 h fermented plant-based protein feed.

**Table 3:** Amino acid profiles (%) of the experimental diets tested in Nile tilapia (*O. niloticus)* reared under biofloc technology (BFT).

Amino Acid (%)	CFM	CPB	FPB6	FPB18
Essential amino acids				
Arginine	1.99	2.35	2.38	2.35
Phenylalanine	1.44	1.87	1.78	1.74
Histidine	0.75	0.94	0.90	0.90
Isoleucine	1.38	1.72	1.66	1.63
Leucine	2.43	3.00	2.89	2.89
Lysine	2.08	2.50	2.28	2.14
Methionine	0.73	0.83	0.90	0.80
Valine	1.58	1.80	1.74	1.73
Threonine	1.04	1.28	1.27	1.24
Non-essential amino acids				
Aspartic acid	3.22	3.87	4.03	3.96
Glutamic acid	5.47	6.52	6.63	6.62
Alanine	1.76	1.71	1.69	1.71
Cystine	0.35	0.46	0.54	0.57
Glycine	2.27	1.56	1.58	1.59
Hydroxyproline	0.46	0.09	0.09	0.10
Proline	1.93	1.98	1.93	1.93
Serine	1.42	1.66	1.70	1.69
Taurine	0.07	<0.01	<0.01	<0.01
Tyrosine	1.05	1.27	1.11	1.22
Sum of amino acids	31.43	35.42	35.09	34.81

CFM—animal protein feed; CPB—plant-based protein feed; FPB6—6 h fermented plant-based protein feed; FPB18—18 h fermented plant-based protein feed.

**Table 4:** Leaching rates (%) of the experimental diets after different times.

	CFM	CPB	FPB6	FPB18
1 min	13.90 ± 2.62 ^a^	14.02 ± 7.16 ^a^	25.04 ± 4.89 ^b^	10.89 ± 1.06 ^a^
3 min	20.80 ± 9.00 ^a^	13.51 ± 2.99 ^a^	38.6 ± 7.92 ^b^	16.59 ± 4.05 ^a^

ND—not detected. Percentages followed by different letters differ (ANOVA, *p* < 0.05) from each other according to Tukey’s test. CFM—animal protein feed; CPB—plant-based protein feed; FPB6—6 h fermented plant-based protein feed; FPB18—18 h fermented plant-based protein feed.

**Table 5:** Shelf life (Log_10_ CFU g^−1^) of each experimental diet.

	CFM	CPB	FPB6	FPB18
Lactic acid bacteria				
0 days	nd	nd	5.77 ± 0.43	5.45 ± 0.30
7 days	nd	nd	5.64 ± 0.54	5.33 ± 0.43
15 days	nd	nd	5.66 ± 0.59	5.06 ± 0.43
30 days	nd	nd	6.63 ± 0.45	5.28 ± 0.41
60 days	nd	nd	4.39 ± 0.56	4.21 ± 0.13
Molds and yeasts				
0 days	6.29 ± 0.52 ^d^	6.09 ± 0.65 ^c^	4.89 ± 1.26 ^a^	4.65 ± 0.56 ^b^
7 days	6.53 ± 0.47 ^d^	6.07 ± 0.10 ^c^	5.35 ± 0.71 ^a^	5.45 ± 0.03 ^b^
15 days	5.39 ± 0.12 ^d^	5.44 ± 0.38 ^c^	3.81 ± 0.16 ^b^	3.07 ± 1.09 ^a^
30 days	6.64 ± 0.57 ^d^	6.61 ± 0.86 ^c^	5.85 ± 0.13 ^b^	4.50 ± 0.70 ^a^
60 days	5.78 ± 0.74 ^d^	5.61 ± 0.13 ^c^	4.47 ± 0.24 ^a^	4.73 ± 0.16 ^b^

Means ± SD followed by different letters differ (ANOVA, *p* < 0.05) from each other according to Tukey’s test. nd—not detected; CFM—animal protein feed; CPB—plant-based protein feed; FPB6—6 h fermented plant-based protein feed; FPB18—18 h fermented plant-based protein feed.

**Table 6:** Microorganism counts in the water system.

	CFM	CPB	FPB6	FPB18
LAB				
0 days	4.36 ± 0.48	4.36 ± 0.48	4.36 ± 0.48	4.36 ± 0.48
7 days	4.05 ± 0.14	4.09 ± 0.31	4.38 ± 0.22	4.47 ± 0.10
15 days	3.46 ± 0.91	3.54 ± 0.62	4.06 ± 0.04	4.31 ± 0.66
30 days	3.68 ± 0.19	3.42 ± 0.75	4.21± 0.07	4.29 ± 0.66
60 days	5.60 ± 0.21 ^b^	5.51± 0.72 ^b^	6.55 ± 0.06 ^ab^	6.65 ± 0.10 ^a^
Heterotrophic bacteria				
0 days	4.74 ± 1.73	4.74 ± 1.73	4.74 ± 1.73	4.74 ± 1.73
7 days	6.38 ± 0.04	5.87 ± 0.94	6.08 ± 0.01	6.00 ± 0.76
15 days	6.24 ± 0.28	6.33 ± 0.66	6.35 ± 0.96	6.56 ± 0.26
30 days	6.20 ± 0.06	5.98 ± 0.53	6.57 ± 0.13	6.27 ± 0.27
60 days	6.02 ± 0.11	5.78 ± 0.13	6.62 ± 0.15	6.34 ± 0.20
*Vibrio* sp.				
0 days	5.25 ± 0.16	5.25 ± 0.16	5.25 ± 0.16	5.25 ± 0.16
7 days	5.35 ± 0.56	5.24 ± 0.72	4.91 ± 0.38	4.17 ± 0.32
30 days	6.93 ± 0.48	6.73 ± 0.50	5.71 ± 1.01	5.85 ± 0.66
60 days	6.89 ± 0.39 ^b^	6.45 ± 0.42 ^ab^	4.89 ± 1.38 ^a^	5.49 ± 0.45 ^ab^

Means ± SD followed by different letters differ (ANOVA, *p <* 0.05) from each other according to Tukey’s test. CFM—animal protein feed; CPB—plant-based protein feed; FPB6—6 h fermented plant-based protein feed; FPB18—18 h fermented plant-based protein feed.

**Table 7:** Performance of juvenile Nile tilapia (*O. niloticus*) fed experimental diets differing in the level of fishmeal inclusion (CFM and CPB) and the fermentation time of plant protein feeds (FPB6 and FPB18) after 60 days of the experiment.

	CFM	CPB	FPB6	FPB18
Weight gain (g)	34.84 ± 7.10 ^a^	22.28 ± 3.18 ^b^	23.44 ± 0.89 ^b^	20.87 ± 1.59 ^b^
Feed consumption (g per fish)	27.50 ± 3.03 ^a^	20.42 ± 1.71 ^b^	30.47 ±1.61 ^a^	28.76 ± 3.14 ^a^
Feed conversion	0.81 ± 0.16 ^a^	0.84 ± 0.02 ^a^	1.28 ± 0.03 ^b^	1.38 ± 0.13 ^b^
HIS (%)	2.11 ± 0.49	1.70 ± 0.32	1.95 ± 0.48	1.68 ± 0.35

Means ± SD followed by different letters differ (ANOVA, *p* < 0.05) from each other according to Tukey’s test. CFM—animal protein feed; CPB—plant-based protein feed; FPB6—6 h fermented plant-based protein feed; FPB18—18 h fermented plant-based protein feed; HSI—hepatosomatic index.

**Table 8:** Intestinal microorganism counts (Log_10_ CFU g^−1^) of juvenile Nile tilapia (*O. niloticus*) fed experimental diets differing in the level of fishmeal inclusion (CFM and CBP) and the fermentation time of plant protein feeds (FPB6 and FPB18) after 60 days of the experiment.

	CFM	CPB	FPB6	FPB18
LAB				
30 days	3.86 ± 0.09 ^a^	3.81 ± 0.39 ^b^	4.39 ± 0.56 ^a^	4.08 ± 0.56 ^a^
60 days	6.18 ± 0.60 ^a^	5.63 ± 0.85 ^b^	6.26 ± 0.69 ^a^	6.44 ± 0.30 ^a^
Heterotrophic bacteria				
30 days	6.51 ± 0.63	6.05 ± 0.36	5.94 ± 0.4	5.75 ± 0.59
60 days	5.25 ± 0.89 ^b^	5.82 ± 0.36 ^b^	6.37 ± 0.33 ^a^	6.00 ± 0.66 ^b^
*Vibrio.* sp.				
30 days	5.45 ± 0.23	5.41 ± 0.31	5.23 ± 0.41	4.14 ± 1.56
60 days	6.80 ± 0.55 ^b^	6.60 ± 0.75 ^b^	5.12 ± 0.70 ^a^	5.30 ± 1.04 ^a^

Means ± SD followed by different letters differ (ANOVA, *p <* 0.05) from each other according to Tukey’s test. CFM—animal protein feed; CPB—plant-based protein feed; FPB6—6 h fermented plant-based protein feed; FPB18—18 h fermented plant-based protein feed.

**Table 9:** Intestinal histomorphometry measurements of juvenile Nile tilapia (*O. niloticus*) fed experimental diets differing in the level of fishmeal inclusion (CFM and CPB) and the fermentation time of plant protein feeds (FPB6 and FPB18) after 60 days of experiment.

Villus	CFM	CPB	FPB6	FPB18
Height (μm)	385.87 ± 38.39 ^a^	268.90 ± 18.19 ^b^	442.60 ± 42.94 ^a^	396.17 ± 30.42 ^a^
Width (μm)	96.86 ± 3.70	86.78 ± 8.38	95.73 ± 21.18	96.3 2± 7.09
Thickness (μm)	47.15 ± 3.51	40.43 ± 4.50	43.46 ± 6.26	42.84 ± 7.12
GCs (u.)	8.69 ± 2.24 ^b^	8.01 ± 2.46 ^b^	16.15 ± 3.75 ^a^	13.82 ± 3.18 ^ab^

Means ± SD followed by different letters differ (ANOVA, *p* < 0.05) from each other according to Tukey’s test. CFM—animal protein feed; CPB—plant-based protein feed; FPB6—6 h fermented plant-based protein feed; FPB18—18 h fermented plant-based protein feed; GCs—goblet cells; u.—each goblet cell was contacted individually.

**Table 10:** Specific activity of selected pancreatic digestive enzymes in the intestinal portion of juvenile Nile tilapia (*O. niloticus*) fed experimental diets differing in the level of fishmeal inclusion (CFM and CPB) and the fermentation time of plant protein feeds (FPB6 and FPB18) after 60 days of the experiment.

Enzyme	CFM	CPB	FPB6	FPB18
Amylase	1.57 ± 1.47	2.09 ± 1.09	1.68 ± 0.47	2.81 ± 0.12
Lipase	3.53 ± 0.26	3.11 ± 0.33	2.58 ± 0.88	2.83 ± 0.96
Total alkaline proteases	1.09 ± 0.24	0.79 ± 0.30	1.04 ± 0.21	0.95 ± 0.19

CFM—animal protein feed; CPB—plant-based protein feed; FPB6—6 h fermented plant-based protein feed; FPB18—18 h fermented plant-based protein feed. Amylase activity was expressed as U mg^−1^ of protein; lipase activity was expressed in U g^−1^; protease activity was expressed as U g^−1^.

**Table 11:** Activity of oxidative stress enzymes in the intestinal portion of juvenile Nile tilapia (*O. niloticus*) fed experimental diets differing in the level of fishmeal inclusion (CFM and CPB) and the fermentation time of plant protein feeds (FPB6 and FPB18) after 60 days of experiment.

	CFM	CPB	FPB6	FPB18
Soluble protein	7.21 ± 2.33	6.98 ± 2.03	9.04 ± 2.73	7.90 ± 2.09
Gluthatione reductase	26.11 ± 8.04	26.77 ± 5.07	21.01 ± 4.51	23.22 ± 5.51
Gluthatione peroxidase	151.48 ± 68.31	147.26 ± 59.75	128.95 ± 56.83	121.32 ± 51.26
Catalase	6.52 ± 9.14	8.82 ± 3.77	7.06 ± 3.37	6.98 ± 2.90
SOD	98.43 ± 0.74	98.70 ± 1.50	99.91 ± 2.01	98.75 ± 2.13

CFM—animal protein feed; CPB—plant-based protein feed; FPB6—6 h fermented plant-based protein feed; FPB18—18 h fermented plant-based protein feed. Protein concentration was expressed as mg mg^−1^; gluthatione reductase, gluthatione peroxidase, and catalase activity was expressed in nmol min^−1^ mg prot^−1^.

## Data Availability

The data that support the findings of this study are available from the corresponding author upon request.
